# Bringing sex toys out of the dark: exploring unmitigated risks

**DOI:** 10.1186/s43591-023-00054-6

**Published:** 2023-03-23

**Authors:** Joana Marie Sipe, Jaleesia D. Amos, Robert F. Swarthout, Amalia Turner, Mark R. Wiesner, Christine Ogilvie Hendren

**Affiliations:** 1 Department of Civil & Environmental Engineering, Duke University, Durham, NC USA; 2 A.R. Smith Department of Chemistry and Fermentation Sciences, Appalachian State University, Boone, NC USA; 3 Department of Geological and Environmental Sciences, Appalachian State University, Boone, NC USA; 4 Research Institute of Environment, Energy and Economics, Appalachian State University, Boone, NC USA

**Keywords:** Microplastics, Nanoplastics, Phthalates, Sex toys, Risk, Consumer products, Vibrator

## Abstract

A majority of American adults report having used sex toys, which, by design, interact with intimate and permeable body parts yet have not been subject to sufficient risk assessment or management. Physical and chemical data are presented examining potential risks associated with four types of currently available sex toys: anal toy, beads, dual vibrator, and external vibrator. A standardized abrasion machine made real-time breakdown of products into microplastics and nanoplastics. The microplastics from the sex toys were then solvent extracted and analyzed using GC–MS. Rates of microplastics and nanoplastics released during abrasion testing from most microplastic release to least was the anal toy, beads, dual vibrator, external vibrator. Both micro- and nanoplastics particles were generated following the abrasion test, with the 50 percentile diameters (D50) ranging from the anal beads at 658.5 μm, dual vibrator at 887.83 μm, anal toy at 950 μm, and external vibrator at 1673.33 μm. The material matrix of each product was analyzed using ATR-FTIR, with results identifying the anal toy as polyethylene terephthalate (PET), the anal beads as polyvinyl chloride (PVC), the external vibrator as a silicone blend (polydimethylsiloxane [PDMS]), and the dual vibrator as a rubber mixture (polyisoprene). After extraction, phthalates known to be endocrine disruptors were present in all tested sex toys at levels exceeding hazard warnings. Analogous findings have been reported for similar materials that, when incorporated into other product categories, are subject to regulatory scrutiny in both the US and EU. This data set is not intended to be representative of sex toys as an entire class of products, nor are the abrasion experiments claiming to simulate exact use conditions. However, these exploratory data frame potential concerns, highlighting research questions and the need for prompt prioritization of protective action. Therefore, future studies and multi-stakeholder action are needed to understand and reduce risk for this class of products.

**Supplementary Information:**

The online version contains supplementary material available at 10.1186/s43591-023-00054-6.

## Background

Consumers expect the products they purchase to be safe for use. When manufacturers fail to protect consumers from avoidable risks, and these failures become known, regulatory agencies are charged to step in with protective action. One class of consumer products for which the evidence of risk is insufficiently understood, communicated, and managed is sex toys which, by design, interact with our most intimate and permeable body parts. While popular journalists and independent activists have called for more transparency of sex toy materials and safety, these efforts must be coupled with scientific investigation to understand and illuminate the risks, and thereby elevate the visibility and priority of these products for risk management actions by manufacturers and regulators.

The intent of this paper is to highlight evidence supporting the possibility that people who use some sex toys in the United States may be exposed to materials that include chemicals previously identified as hazardous, thereby posing potential health risks. We present summarized risk data from the literature as well as our own preliminary data from experiments designed to verify and advance understanding of exposure and hazard in select sex toys. Further, we note comparisons between how the risks of this class of products have been explored and managed with how similar exposures and chemical hazards have been addressed in other less stigmatized products. Finally, we propose a multi-stakeholder, multi-sector collaborative approach to evaluating, communicating, prioritizing, and managing these risks as an effective path to close the current gap between suspected risks and available protections for consumers of sex toys.

### Existing sex toy risk information

#### Known sex toy exposures

Nearly half of U.S. heterosexual men (in a survey of 1047 men aged 18–60) [1] and over half of heterosexual women (in a survey of 3800 women aged 18–60) [2] reported using a vibrator at some point in their lives. Prevalence within the LGBTQ population is higher still, with 70.6% of lesbian women, 79.7% of bisexual women [2], and 78.5% of gay or bisexual men [3] reporting using vibrators and other sex toy products. Thus, consumer exposure to sex toys as a product category is substantial, and unregulated risks could disproportionally affect members of already-marginalized communities. The sex product market is booming, with global sales of sex toys projected to reach $50 billion USD by 2025 [4]. These numbers were amplified during the COVID-19 pandemic as purchases rose compared to the prior year in France (94%), Italy (124%), and Spain (300%) in March 2020 alone [5, 6]. Online sales were also a primary new source of acquiring sex toys and showed a 30% increase reported by *Adam and Eve, one of the largest adult products online retailers* in North America [6, 7]. In addition to online sales, there is also the concern of the contraband market in which sex toys are sold without regulations in places like Thailand, Saudi Arabia, India and throughout Africa [8]. Considering growing consumer access to and increased popularity of sex toys, coupled with the expanding options on the market, it is likely that consumer exposure to unregulated risk will continue to increase over time.

#### Known sex toy hazards

The documented physical, biological, and chemical hazards of sex toys can produce a range of acute and chronic health effects. Given that products manufactured for recreational sexual use comprise a wide variety of materials and forms, most ostensibly intended for contact with sensitive body parts, assessing associated risks is a daunting task. Though we have purposefully limited our scope to omit treatment of potential social-emotional issues, the preliminary data suggest that sex toy bodily hazards are substantial [9]. Between 1995 and 2006, 6,799 adults over 20 years of age sought ER care in the United States for sex toy-related injuries, with reported injuries rates dramatically increasing in the last six years of the study [10]. The true incidence of sex-toy-related injuries is likely higher because negative stigma leading to embarrassment can cause underreporting and delays in seeking treatment. Such delays can increase complications, including infection-related deaths. Chemical hazards include short- and long-term health effects associated with a range of toxic chemicals identified in sex toys; available research on sex toy chemicals has emphasized phthalates, a family of synthetic chemical compounds that increase the flexibility of plastics [11]. While phthalate effects on human health endpoints are not well studied, several phthalates have been identified as toxic in animals, with phthalate exposures resulting in a suite of reproductive, developmental, and carcinogenic effects targeting the liver, testes, uterus, ovaries, thyroid, and developing fetuses [12, 13]. One common phthalate, diethylhexyl phthalate (DEHP), is categorized as toxic by the U.S. Consumer Product Safety Commission (CPSC) within the Federal Hazardous Substances Act (FHSA). Prior material analyses of sex toys like those characterized here revealed phthalate concentrations in most tested products at concentrations ranging from 24–60% by weight [11, 14, 15]. In addition, there is growing concern over human exposure to micro-and nano-plastics. The translocation and biouptake of nano-sized particles is now well established [16]. Human exposure to nanoplastics and the potential for enhanced release of plastic additives are of potential concern [17].

### Aggregating and expanding the current state of sex toy risk knowledge

**Fig. 1:**
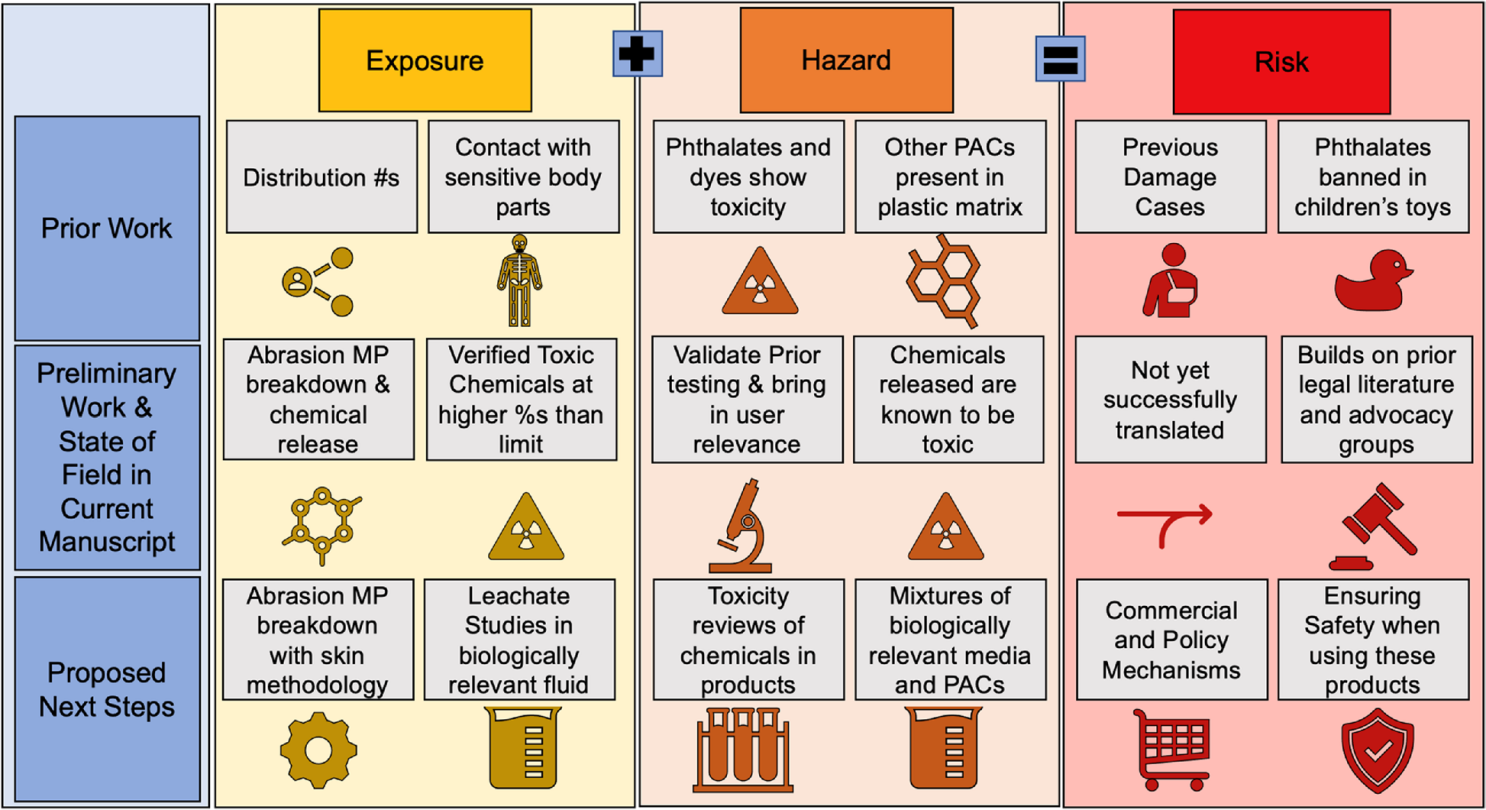
Sex Toy risk schematic: current state of exposure, hazard, and risk knowledge. [PAC = Polymer Additive Chemicals; MP = Microplastics]

The current study summarizes known data from existing studies and adds additional experimental data to evaluate prior claims and identify knowledge and action gaps. This paper applies risk forecasting expertise developed within the convergent field of nanomaterial environmental health and safety as well as the emerging nano- and microplastic field to evaluate exposure scenarios for sex toys. Four different models of sex toys were purchased for analysis and characterization; they were selected for comparison with prior results from the literature where possible, as well as to represent a range of use categories. The studied devices include: a dual vibrator (Deluxe Rotating Wall Bangers Rabbit vibrator), anal beads (Cal Exotics X-10 Beads Blue), and an anal toy (Stubby Nubby G-vibe pink). These three model types were selected to recreate or to purchase as close as possible an analog of the sex toys investigated in the Tønning et al., Nilsson et al., and California phthalate reports [11, 15, 18]. The fourth product, an external vibrator (Luna rechargeable personal massager), was selected for comparison due to its popularity on Amazon.com combined with the packaging claims that it was made from “medical grade silicone”. All products were purchased in triplicate, with each of the three samples purchased at different times to account for sample and batch variability. This data set is not intended to be exhaustive or fully representative of the sex toys as an entire class of products; however, it frames the potential magnitude and nature of concerns, highlighting research questions and the need for prompt prioritization of protective action. A summary of prior and preliminary data on sex toy exposures and hazards demonstrates that the potential health risks associated with these products may be substantial and require further investigation (Fig. 1). Through comparison with health risk data for other products for which safety standards have been adopted, we suggest areas of needed research to enable multi-stakeholder action toward mitigating sex toy risks.

## Materials and methods

### Materials

Four different models of sex toys were purchased for analysis and characterization. They were selected for comparison with prior results from the literature wherever possible, as well as to represent a range of use categories. The studied devices include: a dual vibrator (deluxe rotating wall bangers rabbit vibrator), an external vibrator (Luna rechargeable personal massager), anal beads (Cal exotics X-10 Beads Blue), and an anal toy (stubby nubby G-vibe pink). These products were purchased in triplicate, with each of the three samples purchased at different times to account for sample and batch variability.

### Abrasion method

An abrasion machine was used to calculate fragmentation rates of the materials making up these internal-use products. This study was done to examine the exposure potential of these products for nano- and microplastics using a standardized method. A custom abrasion setup was created as previously described in Sipe et al. [19] and is briefly summarized here. A weight presses down on a rod, providing a normal force on the plastic sample attached to the bottom of a plate. The power input to the abrasion process can be characterized in a scalable fashion through knowledge of this normal force and the torque measured on the rotating abrading element. The sample is pressed onto the abradant, in this case a glass file, which rotates and is enclosed in the space which allows for abraded nano- and microplastics to be collected for further analysis. Abrasion rates were measured by weighing the sample before and after thirty seconds of abrasion, changing the weight applied to the sample (from 0.1 to 2 kg), and keeping speed constant at around 800–1000 rpm as a standard start rate. After cleaning out the inside of the machine and placing the machine under a hosed fume hood, collectors were placed to recover the abraded nano- and microplastics. To avoid external contamination, the lab users wore cotton lab coats and clothing, regularly cleaned the chamber with cleanroom wipes and ethanol after each sample and stored each sample in a glass container. The glass file was sanitized with ethanol after every test to preclude contamination. Power input was calculated using the value of torque measured (Datum electronics, M425, Isle of Wight, UK). Abrasion rates were measured over a range of power input from 0 to 200 W.

### Nano- and microplastic characterization

Nano- and microplastics were obtained for image analysis. For microscope images, MP particles were put between glass slides and a black background. They were imaged using a Nikon Eclipse E-600 compound byte using a Nikon DXM 1200 Digital Camera and NIS Elements 3.1 software at 2 × magnitude. The Mastersizer 3000 (Malvern, UK) was used to calculate the average plastic particle size from the abraded results. The particles were suspended in ethanol to ensure their dispersion through the device. MP particles were suspended in DI water and shaken for one month in glass vials, and then measured for their dispersion into nanoplastics. These dispersions were then measured using a Nanosizer NanoZS (Malvern Panalytical Ltd.).

### Identification of plastic matrix

Fourier-transform infrared (FTIR) spectroscopy was used to identify the material matrix of each sex toy. A Thermo Electron Nicolet 8700 FTIR spectrometer was used in attenuated total reflection (ATR) mode through the Shared Materials Instrumentation Facility (SMiF) at Duke University. All spectral data samples were collected and processed using Thermo Electron’s OMNIC operating software system. Samples were analyzed before and after the phthalate extraction protocol using a germanium crystal over the spectral range of 5500 – 650 cm^−1^. Two libraries were used to compare spectral matches identifying the toys’ matrices: the Hummel Polymer Sample Library accessed through the OMNIC system, and the Open Specy’s publicly available microplastics library [20].

### Phthalate analysis methodology

#### Standard solutions

All solutions were prepared gravimetrically. All glassware was precombusted in a muffle furnace at 450 °C for 8 h. A multicomponent phthalate ester standard stock solution containing all analytes listed in EPA Method 8061A was used to create a seven-point calibration curve ranging from 0.05–140 ppm. Each calibration solution was amended with approximately 1 ppm of benzyl benzoate as an internal standard (Restek) and dibenzyl phthalate, diphenyl isophthalate, and diphenyl phthalate as surrogate standards (Restek).

### Sample preparation and extraction

Triplicate samples of the outer surface of each toy were cryomilled. Approximately 100 mg of each cryomilled sample was spiked with surrogate standard at a concentration equivalent to the calibration standards. The silicone sample (Luna) was extracted using 8 mL of environmental grade dichloromethane (DCM) using a vortex mixer. Similarly, the other samples (Beads, Dual vibrator, Anal Toy) were extracted using environmental grade hexane and HPLC grade methyl ethyl ketone (MEK) following the protocol of Krongauz et al. (2014) [21]. Briefly, the sample was extracted with approximately 10 mL of 1:2 MEK and hexane using a vortex mixer. All samples were gravity filtered through precombusted GF/F filters and the sample extracts were washed with 0.9% saline solution and 1 g NaCl (s). Internal standard was added to each sample prior to GC–MS analysis using the same target concentration as in the calibration standards.

#### Quality control

Solvent blanks, laboratory reagent blank (LRB) samples, and laboratory fortified blanks (LFB) were run alongside the samples. The LRB samples were made using the protocols for silicone and PVC samples without addition of any toy material. The LFB samples were prepared at a concentration of EPA 8061A standard solution of 10 ppm and extracted using the methods for silicone and PVC extraction. Surrogate and internal standards were added as described for sample preparation and extraction.

#### Instrument

An Agilent 6890N gas chromatograph (GC) with a 5973 inert mass selective detector (MS) was used for analysis. The GC was equipped with a DB-5MS column (30 m × 0.25 mm × 0.25 μm; Agilent). The oven parameters were as follows: 60 °C for 0.50 min, an initial ramp of 25 °C/min until 160 °C, a ramp of 15 °C/min until 320 °C, and holding for 1 min. All samples were run splitless with a 1.0 uL injection and helium carrier gas. The MS was operated in selected ion monitoring mode. Extracted ion chromatogram peak areas for each phthalate in the Restek EPA 8061A mix were normalized to the response of the internal standard and quantified using the respective calibration curve except for diisononyl phthalate (DINP) and diisodecyl phthalate (DIDP) which were determined using the calibration curve for Di-n-nonyl phthalate (DnNP) because these phthalates are not included in the EPA 8061A method. All reagent blanks were free of analytes. Surrogate standard recoveries ranged from 95–103%, and percent recovery of analytes in the LFB ranged from 92–110%.

## Results

### Sex toy abrasion

**Fig. 2:**
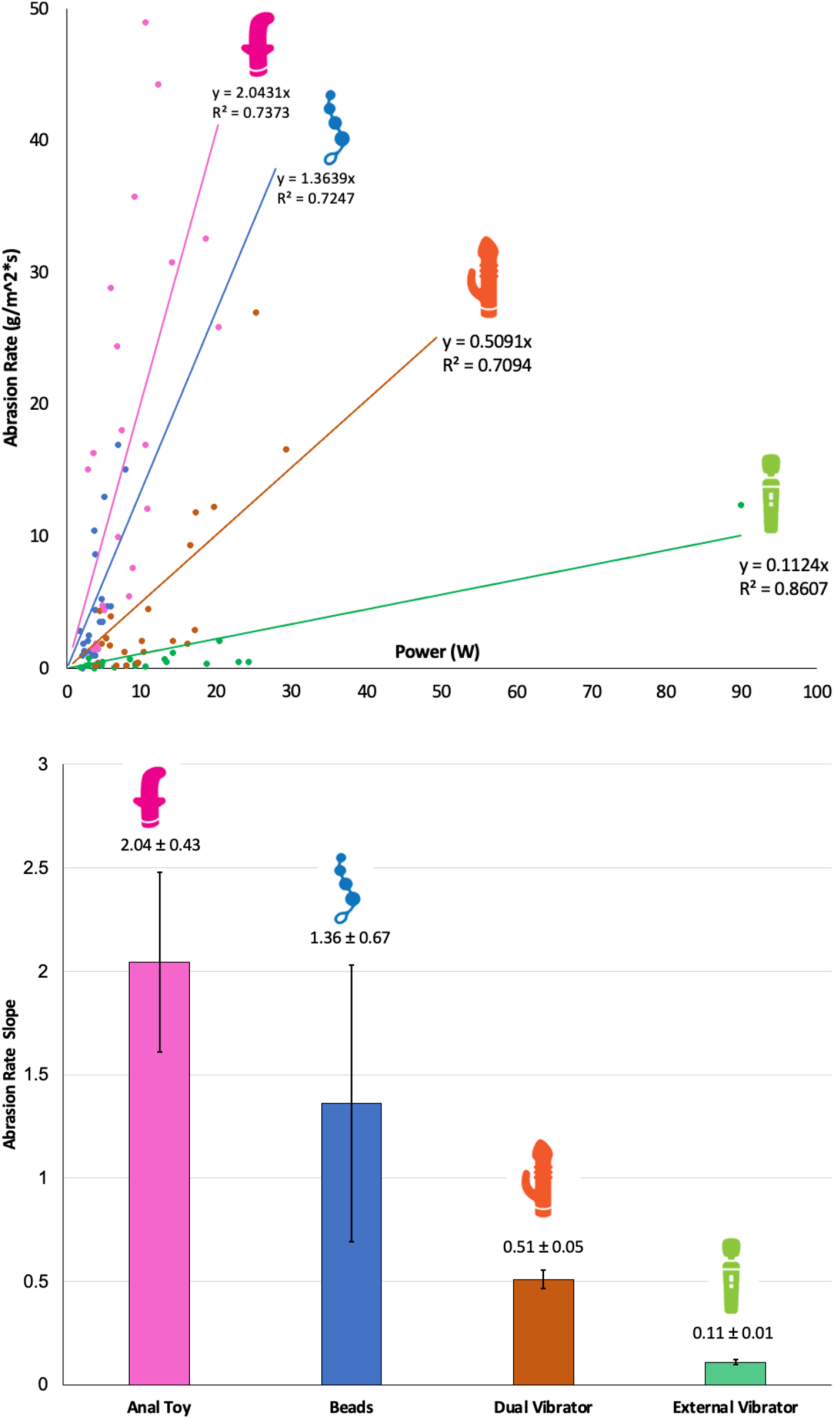
Abrasion of Sex Toys. (Top), and Abrasion rate as a function of power applied to material (Bottom) comparison of the relationship between abrasion rate and power for each material. Error bars represent the 95% confidence intervals of the slopes of the linear regression lines shown in the left panel

Abrasion was applied to test whether breakdown would be induced using a standard method developed for consumer product testing, with linear model fits. To simulate abrasion conditions representative of actual product use, further methods development and studies will be required; this initial standard method was applied as proof of concept, in line with work on other product matrices. Using methods and equations from Sipe et al. and Bossa et al. [19, 22], the abrasion rate was calculated in units of grams released per surface area of product tested over time (Fig. 2). The anal toy displayed the highest abrasion rate followed by the beads, then the dual vibrator, and lastly the external vibrator. Figure 2 also shows these slope value abrasion quantities as bar graphs with standard error on the slope. All materials had R^2^ values over 0.7. The confidence intervals on the slope estimate that the anal toy was not significantly different than the beads. The dual vibrator had the third the greatest number of microplastics produced and lastly the exposure to the production of microplastics from the external vibrator was the least of the four sex toys. These results confirm differences in potential microplastic and nanoplastic generation from abrasive forces applied to sex toys. This breakdown rate, while potentially higher than it could be in actual use scenarios, provides understanding of a potential upper bound in mechanically induced exposure. Exposure in this study will reference the generation of microplastics from the material in which the sex toys are generated from as well as the phthalates the material contains, raising the possibility of interaction with these microplastics and chemicals generated.

### Sex toy microplastic characterization

**Fig. 3:**
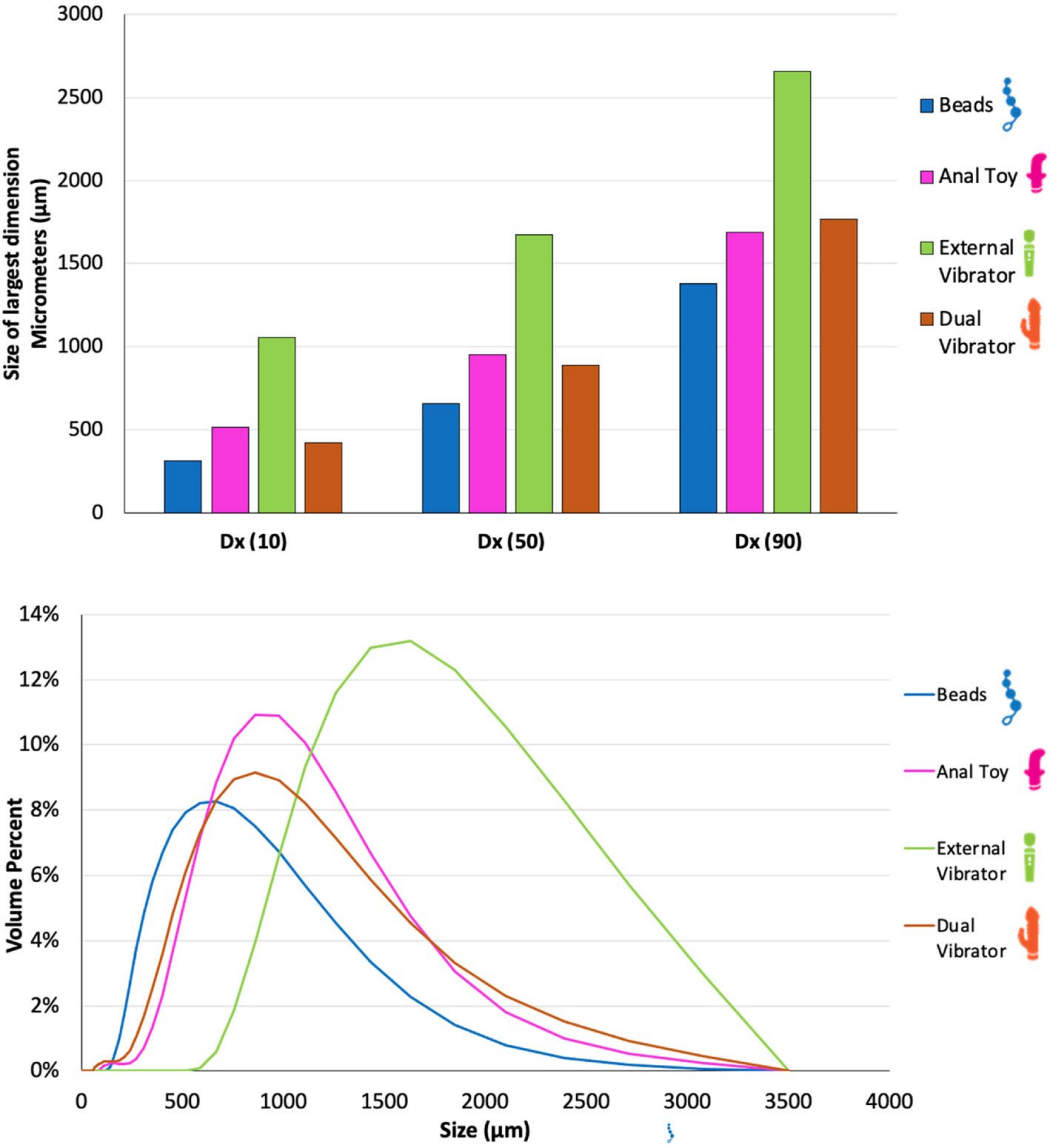
Size Distribution of microplastics from abrading Sex Toys. (Top) D10, D50 and D90 and Size distribution (Bottom) Volume Particle Distribution in micrometers percentage

Microscopic images show the relative size distribution and shape of the microplastics generated during mechanical abrasion in terms of volume (Fig. 3). The distinct results amongst the four types of plastic products tested show that the microplastics produced are more a function of the material and how it is produced rather than an artifact of the abrasion machine. The 50 percentile diameters (D50) of the microplastics generated from sex toys (from smallest to biggest) were beads at 658.5 µm, dual vibrator at 887.83 µm, anal toy at 950 µm and external vibrator at 1673.33 µm. These values are shown below in Fig. 3. Although the external vibrator’s D50 was above 1 mm, smaller microplastics were abundant in the abraded samples. Even smaller particles were subsequently generated from microplastic suspensions for each sex toy tested after one month of microplastic breakdown in DI water. These secondary particles had D50s of 432 ± 0.43 nm for suspensions of microplastics abraded from the beads, 1066.87 ± 1.07 nm for the external vibrator, 1605.63 ± 1.61 nm for the anal toy, and lastly 1714.67 ± 1.71 nm for the dual vibrator.

**Fig. 4:**
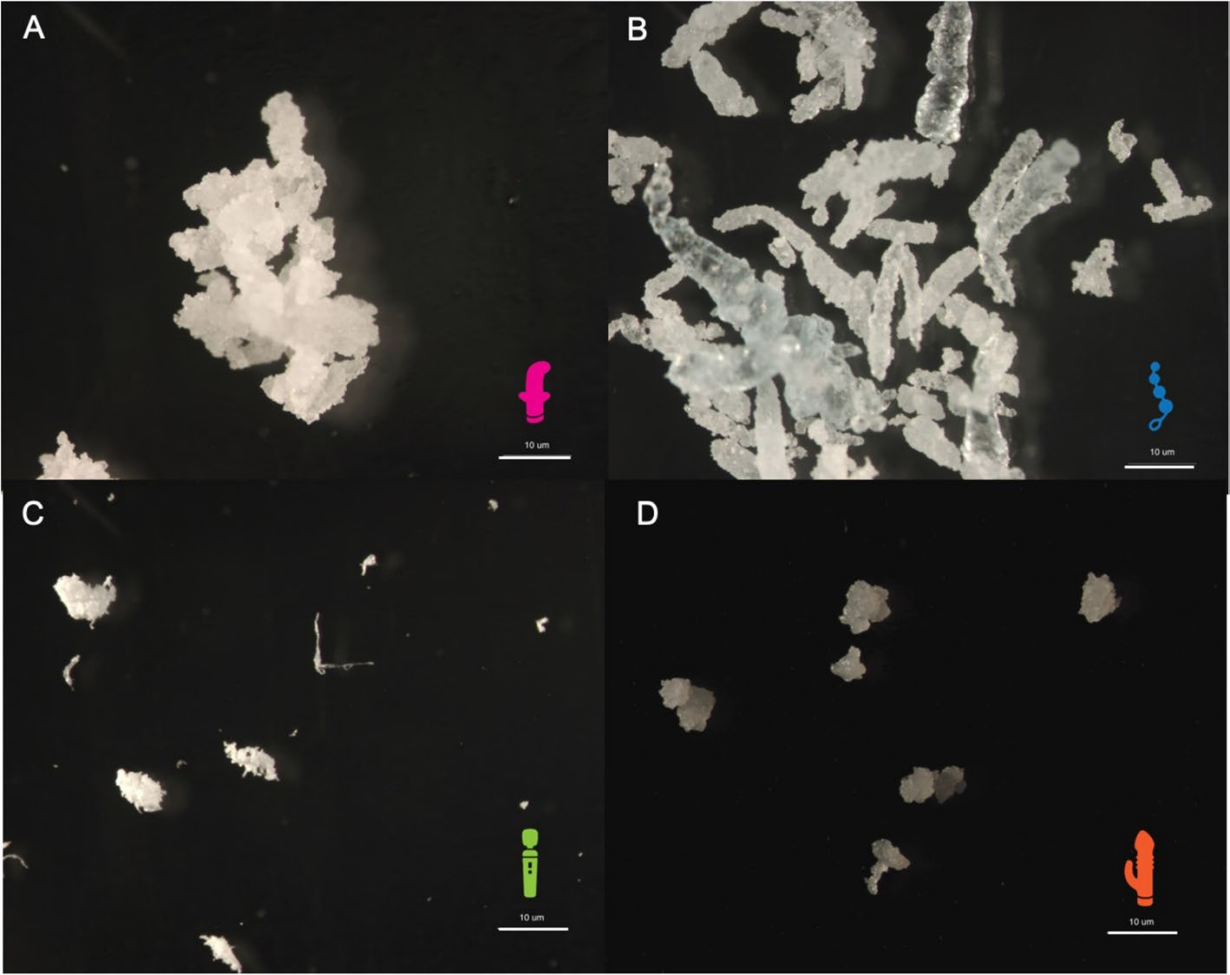
Sex Toy microplastic microscope images all scale bars read 10 µm. **A** Anal Toy **B** Beads **C** External Vibrator **D** Dual Vibrator

Compared with the surface area of the initial toy, the abraded materials present high specific surface areas that may facilitate transport of additives from the material. Images of the abraded microplastics are shown below in Fig. 4.

#### Gas chromatography/mass spectrometry phthalate analysis

Numerous phthalates were found in all four sex toys sampled in concentrations spanning over three orders of magnitude (Table 1). The external vibrator contained the greatest number of individual phthalates with 7 different phthalates detected but at low concentrations. Both the dual vibrator and beads only had di-n-octyl phthalate (DnOP) present but at concentrations higher than the 0.1 weight % allowed in children’s toys by US regulatory agencies. All four sex toys contained phthalates that are either over the 0.1 weight % limit (for DnOP) or banned under REACH ((Registration, Evaluation, Authorization, and Restriction of Chemicals) by the ECHA (European Chemicals Agency) in the EU (European Union) (DEHP, BBP and DBP) [23]. For clarification, this applies to children’s toys and is not illegal to sell currently as sexual wellness products.

**Table 1:**
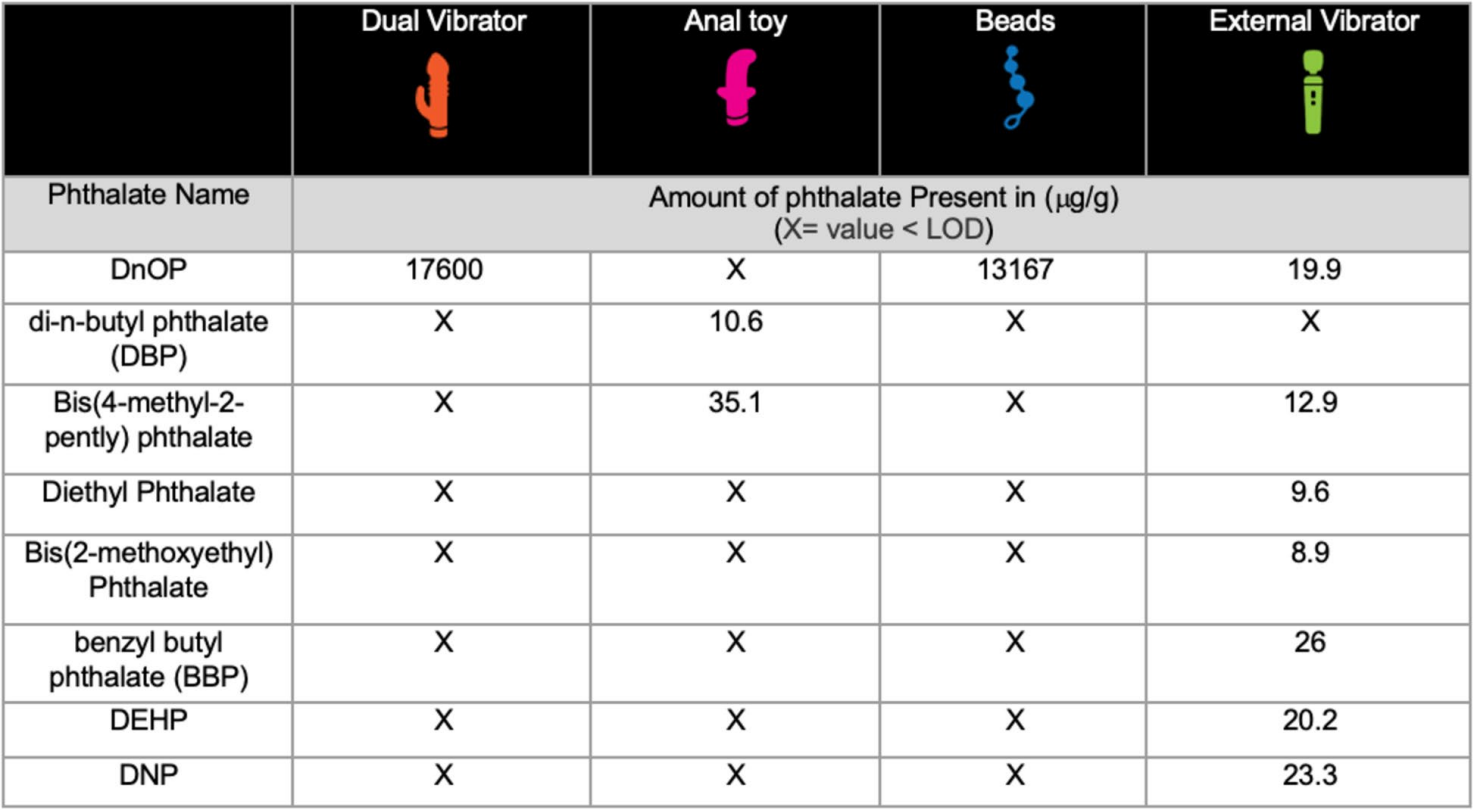
Phthalate presence in sex toys

### Identification of plastic matrix

The toys’ matrices were analyzed to verify the manufacturers’ self-reported matrix on the external packaging. ATR-FTIR analysis of the toys’ matrices was performed before and after phthalate extraction (Figure S1-S3). However, the anal toy was extracted with dichloromethane (DCM) for spectral analysis since the phthalate extraction fully degraded the matrix (Figure S4). Initial ATR-FTIR spectral analysis mis-identified all the toys as polyethylene terephthalate (PET) due to strong absorbance between 960 – 990 cm^−1^, which was believed to represent the phthalates present in the toys. Differences in abrasion rates and observed toy textures indicated that the toys’ matrices were not identical to each other. ATR-FTIR spectral analysis after the phthalate extraction protocol indicated that initial assumptions about the phthalate peak were likely. Once the peak between 950 – 990 cm^−1^ was removed, the toys’ matrices were matched between two spectral libraries: OpenSpecy and the Hummel polymer and additive library [20]. Resulting tests of the matrix composition determined that the anal toy was polyethylene terephthalate (PET), the anal beads were identified as polyvinyl chloride (PVC), the external vibrator was a silicone blend (polydimethylsiloxane [PDMS]), and the dual vibrator was a rubber mixture (polyisoprene). The dual vibrator was the only toy not to specify the material matrix of the toy. All the other toys’ identified matrix matched the self-reported material matrix. It is important to highlight that these sex toys are comprised of highly complex matrices containing additives, that include phthalates and dyes, which hindered matrix identification. The matrices of these toys were only able to be identified once some of the additives were removed.

## Discussion

Our studies indicate that sex toys can break down into microplastics and the materials contain phthalates that have been associated with health concerns. A summary of risk-relevant information explored and compiled in this study is shown below in Table 2. The compilation of information presented in Table 2 represents a subset, not an exhaustive list, of all potential risks or exposures. Similarly, the work presented here does not claim to represent a risk assessment of sex toys as a class of products; rather, this synthesis and presentation of new data is presented to demonstrate potential for exposure based on the observed presence, and demonstrated release, of substances that have been tested by others and found concerning or definitively hazardous. These data support that microplastics were generated as the product was abraded using a standard product abrasion method and indicate that some fraction of nanoplastics were present in every sex toy sample tested, suggesting the potential for exposures to hazardous materials under conditions of abrasion. The rate of the breakdown of these materials from the products is also important to note, since if material breakdown does end up being demonstrated to occur under realistic use conditions in the future, this could indicate that resulting materials might directly be in contact sensitive tissues since this class of products is specifically designed for use scenarios that involve sensitive body parts. Also of utmost concern is the presence of phthalates in concentrations exceeding US regulatory and EU regulatory standards for similar products. To understand if this concern is warranted, again further studies would be required to test whether the risk scenario represented by an infant mouthing a plastic toy is indeed similar to the use of a sex toy that may be comprised of the same product plastic matrix, with the same phthalate additives, and also in contact with a mucous membrane. We stress that we have not attempted to prove this is equivalent in the current work. Rather, we assert that since the measured presence of phthalates in our small sample size exceeds the exposure limit for the same chemicals in the US Consumer Product Safety Commission (CPSC) regulations in children’s toys of greater than 0.1% of the product weight, investigations into whether or not the risk scenarios are also similar, are prudent for public health protection [24]. Some of the phthalates identified in our experiments have been observed concurrently with serious fertility complications or loss of fertility in rodents at high concentrations; though causation may not have been demonstrated, the correlation is concerning enough to warrant further investigation. Additionally, significant toxicity of phthalates to aquatic organisms has been reported by ECHA [23].

**Table 2:**
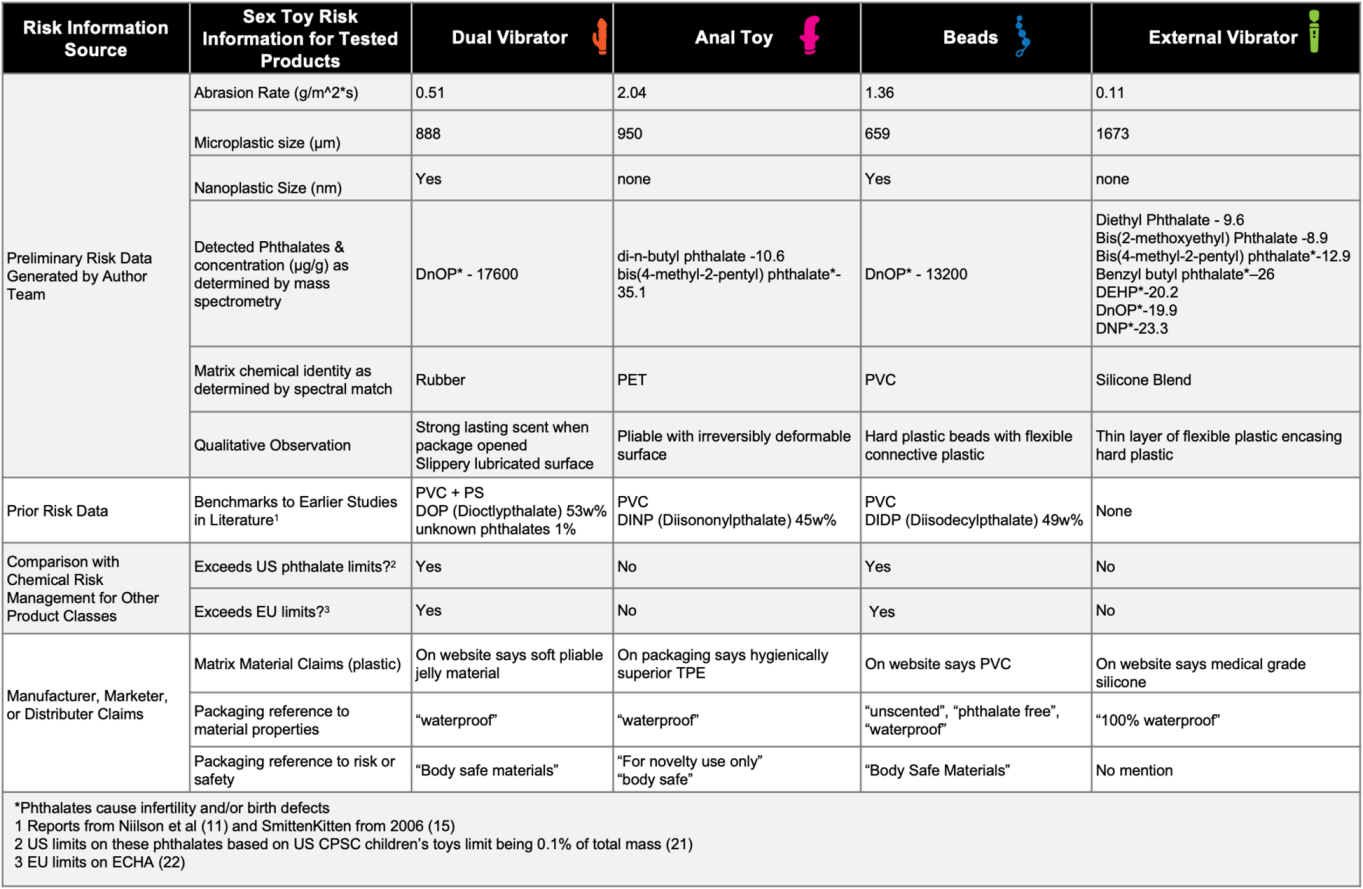
Conclusions of study

The US CPSC restricts levels of eight phthalates in children’s toys and childcare objects to less than or equal to 0.1% of the mass of the plasticized material. Toys and childcare products are expected to be mouthed by babies and in contact with the oral mucosa of children for prolonged periods of time, and substantial exposures to phthalates could occur from the normal use of these products by children. While children carry higher metabolic concentrations of phthalates, adults could potentially still be affected [25]. However, when the CPSC published external reviews of exposure data for select phthalates [24], these reviews were only concerned with children’s toys. Therefore, the external reviews did not include any US-based chemical studies on sex toys. A comprehensive investigation of adult exposures to phthalates drawing on all possible datasets should be considered as part of understanding any potential exposure to phthalates from sex toys in a broader context (e.g. CDC Calafat and NHANES dataset) [26, 27]. For example, NHANES data from 2009–10 offer data on phthalate metabolites based on urine samples. A targeted, funded risk assessment of sex toys as a class of products could interrogate whether these and other datasets may offer relevant insight to guide consumer protection. The EU restricts three types of phthalates – or any mixture thereof—to 0.1% by mass in children’s products likely to contact the mouth, while a further four are banned in all articles with expected prolonged contact with human skin (i.e., 10 min or more) [23]. REACH restrictions through ECHA restrict phthalates that are classified as toxic to reproduction in restriction in products since 2006. Through this ruling, ECHA restricted any products that contain DEHP, DBP, DIBP and BBP in expectance “to save 2,000 boys each year from impaired fertility later in life” [23]. Although the dataset is small, the phthalate concentrations found suggest that high exposures to these toxic compounds are possible for the large segment of the population that uses sex toys.

Beyond the chemical and physical hazards present, risk to consumers is also enhanced through inaccuracies in marketing communication. Sex toys are made of material matrices that are in some cases presented to the consumer incorrectly; for instance, one studied in our preliminary testing here was overtly advertised as being “phthalate-free” when chemical analyses revealed this was not the case. Another toy was sold with package messaging identifying it as “novelty gag gift not intended for safe use”, even though the other side of the same packaging contained the label “body safe”. Companies currently use labels such as “for novelty use only”, with the result being they avoid FDA regulation as medical devices. These labels are directly reported from packaging for the sex toys our team shopped for and purchased for these studies. Although they do not represent a complete marketing and communication study, they clearly illuminate the possibility that overall, players across the supply chain for sex toys are not being held accountable for accurate package labeling regarding chemical or physical safety for bodily use.

Additional complexity is introduced in considering the interactions between different attributes of product use scenarios, requiring analysis of properties of the products themselves together with relevant surrounding media, mechanical forces, and biological factors. For example, exposure studies for risk of 16 sex products were analyzed for potential risk as shown in Tønning et al., in which they demonstrated that lubricants facilitated higher transport of DEHP, with migration of DEHP from one product into water- and oil-based lubricants approximately 7 × and 1000 × higher, respectively, than in artificial sweat [11, 18]. The surrounding media properties in these exposure studies mimic use conditions for many of these products, highlighting that the context of various bodily fluids may influence the transport and availability of concerning chemicals. Future studies should incorporate real or synthetic bodily fluid leachate tests along with realistic abrasion conditions. Other compounded risks may require examination as well; nano-scale fractions of abraded materials could be worth future investigation, as particles in this size range may in theory also be small enough to travel across biological membranes, where additives such as phthalates may subsequently diffuse out of the plastic matrix and into the body depending on physical–chemical gradients in the surrounding fluid. The exploratory studies presented here, in conjunction with existing data synthesized for this manuscript, illustrate that sufficient questions persist regarding the potential for release of concerning materials from sex products to justify targeted investigations into potential exposures and hazards specific to this class of consumer products.

Jurisdiction for sex toy policies and necessity to investigate risks.

Accurate and shared product classification could provide context for further research and for mitigation of sex toy risks in the US. Though sex toys currently fall within the product jurisdiction of the CPSC, to date the agency has not imposed labeling or invoked analogous materials standards within this class of toys as it has within children's toys of the same chemical make-up. The US Food and Drug Administration (FDA) classifies vibrators as “obstetrical and gynecological therapeutic medical devices for treating sexual dysfunction and improving pelvic floor muscle tone.” Although there is a need for therapeutic devices in protecting pelvic floor health, many people do not purchase sex toys with this intended use. The history of vibrator development in the 1880s for clinical use to stimulate women as a medical treatment for “hysteria” is a matter of current scholarly debate [28, 29], but the classification of vibrators as medical devices has persisted. Manufacturers avoid regulation under FDA medical device jurisdiction by including “for novelty use only” on labels, implying that the product is intended as a “gag gift” with no practical use. However, this disclaimer does not reflect consumer use patterns and has little legal meaning. Just as flexible plastic children’s toys can be put into children’s mouths, creating an exposure pathway to hazardous chemicals, the materially identical class of toys manufactured for adult sexual contact will interact with sensitive body parts, creating similar exposure pathways and consumer harm. This ruling was made in context of infants’ cumulative exposure to via other pathways; a comprehensive risk assessment of potential exposures from sex toys would similarly require assimilation of sex toy specific data into the broader context of exposure data for adults as advocated for earlier in the discussion. The good news for consumers is that this class of devices can be collectively prioritized for additional research and protective action. Sex toys, like any other toy, are consumer products with established paths available to improve labeling and product safety, including the elevation of priority through public awareness.

### Voices needed in the multi-disciplinary, multi-sector effort to address safety of sex toys

**Fig. 5:**
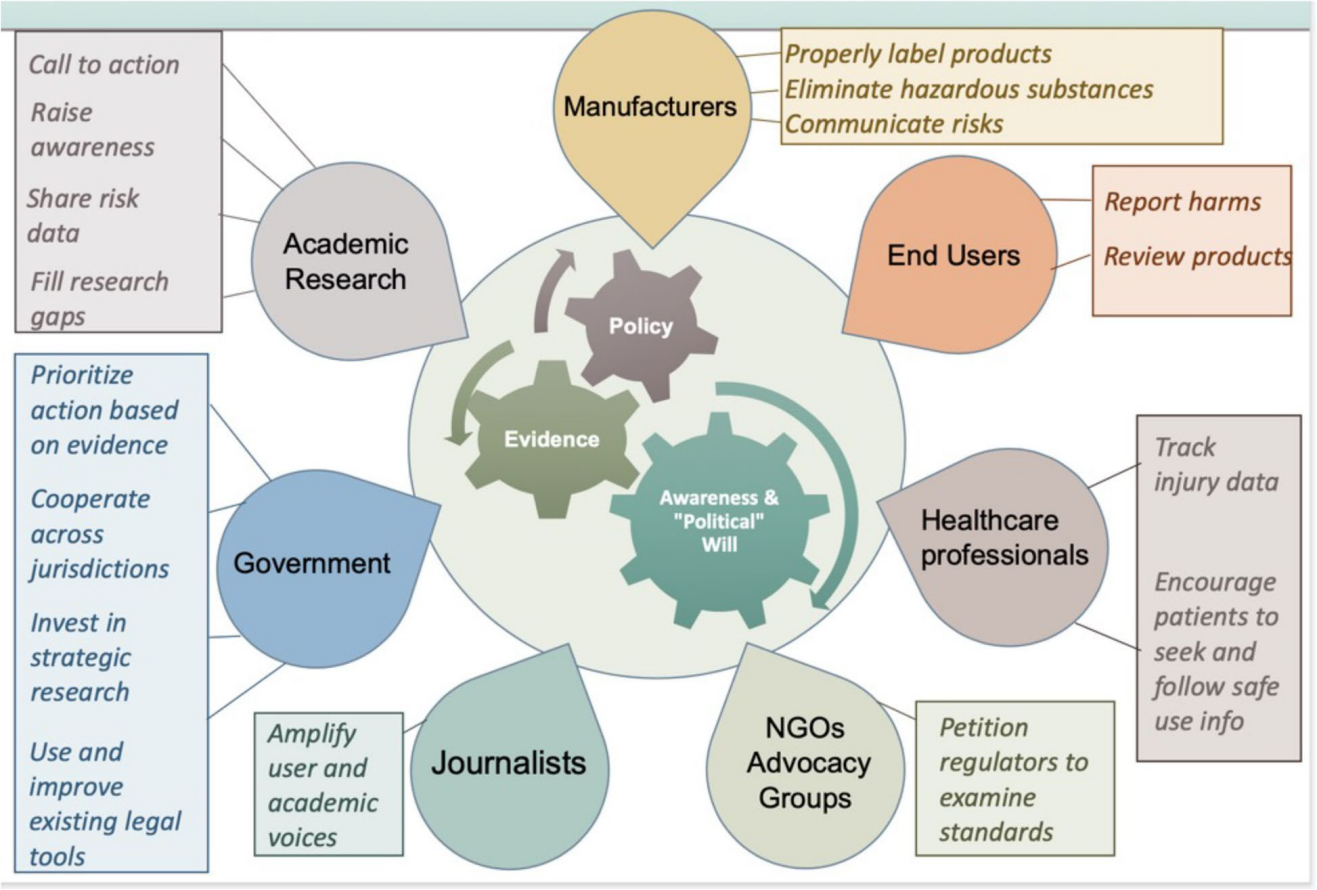
Multiple stakeholder roles are required to address sex toy health risks

The work of protection and risk communication around sex toys in the US has thus far largely occurred within self-assembled consumer and sex toy industry stakeholder groups, potentially due to the societally taboo nature of the products. Popular culture articles [30], sex toy critics [31], and sex shops [15] have raised awareness of chemical hazards, and healthcare professionals have called for educating consumers about preventable physical injuries associated with poorly designed sex toys [32, 33]. Legal scholars have brought attention to the absence of regulatory action by the U.S. federal government to address these risks and have called upon consumer action groups – and even the US Congress – to step in and demand protective action [9, 34] but have so far gained little traction. In addition, risk scholars have addressed the heuristic biases that can be introduced when a source of cultural hesitance, such as a reaction to private or taboo topics, causes an impediment of forthright risk assessment and management [35, 36]. Such objective analysis is needed now, as the landscape of sex-related products will only become more complex with the convergence of artificial intelligence, advanced materials, robotics [37] and indeed sex robot brothels [38]. We must address the current more straightforward class of sex toys as a start, and a first step will be investing in research to corroborate risk evidence and motivate protective action. This requires the work of multiple stakeholders to address this risk and advocate for transparency and regulations for safety of the affected demographics, shown in Fig. 5.

The authors intend this manuscript to help elevate this issue through the contribution of preliminary studies conducted from a risk scholarship perspective, drawing upon approaches developed in the nanoEHS community and nano- and microplastics exposure and hazard forecasting. Critical next steps include the development of a standardized method for microplastic, nanoplastic, and chemical exposure during use of products such as these representative sex toys, including improved simulation of dermal and internal exposure abrasion mechanisms. This study also illuminates the need for a more representative encompassing exploration of microplastic and chemical exposure from sexual wellness products and feminine products, expanding product selection beyond those in this initial study and Tønning et al. to generate a broader range of results representative of increasingly realistic exposure scenarios [18].

Increased awareness of potential risk can catalyze a broader conversation around protecting consumers from sex toys risks, support advocacy for additional research, and promote stakeholder involvement to champion protective action. Consumers and organized stakeholders can play a key role in influencing the priorities of government regulatory agencies by reiterating the need for research efforts through public engagement pathways. Representative inclusion across all genders and sexual orientations will be important to ensure attention to the risks accrued to, and protections needed by, key affected demographics. Straightforward, evidence-based insistence on quantitative assessment of the risks can overcome cultural hesitance to addressing this sensitive topic and motivate the research necessary to enable regulatory agencies to take appropriate action.

## Supplementary Information

**Additional file 1.**

## Data Availability

All data are available in the main text or the supplementary materials.

## Abbreviations

CPSC: US Consumer Safety and Product Commission
DBP: Dibutyl Phthalate
DEHP: Diethylhexyl phthalate
DIBP: Diisobutyl Phtalate
DIDP: Diisodecyl phthalate
DINP: Diisononyl phthalate
DnOP: Di-n-octyl phthalate
ECHA: European Chemicals Agency
EU: European Union
ATR-FTIR: Attenuated Total Reflection Fourier-transform infrared Spectroscopy
GC–MS: Gas chromatograph mass spectroscopy
LFB: Lab fortified blanks
LRB: Lab reagent blanks
nanoEHS: Nano Environmental Health and Safety
PDMS: Polydimethylsiloxane
PET: Polyethylene Terephthalate
PVC: Polyvinyl Chloride
REACH: Registration, Evaluation, Authorization, and Restriction of Chemicals
SEBS: Styrene-ethylene-butylene-styrene

## References

[1] Reece M, Herbenick D, Sanders SA, Dodge B, Ghassemi A, Fortenberry JD (2009). Prevalence and characteristics of vibrator use by men in the United States. J Sex Med.

[2] Herbenick D, Reece M, Sanders S, Dodge B, Ghassemi A, Fortenberry JD (2009). Prevalence and characteristics of vibrator use by women in the United States: results from a nationally representative study. J Sex Med.

[3] Rosenberger JG, Schick V, Herbenick D, Novak DS, Reece M (2012). Sex toy use by gay and bisexual men in the United States. Arch Sex Behav.

[4] Frost, Sullivan. Femtech—Time for a Digital Revolution in the Women's Health Market. Frost & Sullivan. https://www.frost.com/frostperspectives/femtechtime-digital-revolution-womens-health-market/. Published May 25 2021. Accessed 8 Feb 2023.

[5] Dickson E. Thanks to COVID-19, internet-connected sex toy sales are booming. Rolling Stone. https://www.rollingstone.com/culture/culture-news/teledildonics-remote-sex-toy-salescovid19-coronavirus-pandemic-975140/. Published March 2020, 31.

[6] Arafat SMY, Kar SK (2021). Sex during pandemic: panic buying of sex toys during COVID-19 lockdown. Journal of Psychosexual Health.

[7] Drolet G. Sellers of sex toys capitalized on all that alone time. Manhattan: New York Times. 2020. https://www.nytimes.com/2020/06/07/style/sex-toys-online-coronavirus.html.

[8] Naik Y (2021). Regulations on sex toy industry in Europe Law. Technium Soc Sci J.

[9] Stabile E (2013). Getting the government in bed: how to regulate the sex-toy industry. Berkeley J Gender L Just.

[10] Griffin R, McGwin G (2009). Sexual stimulation device-related injuries. J Sex Marital Ther.

[11] Nilsson NH, Malmgren-Hansen B, Bernth N, Pedersen E, Pommer K. Survey and health assesment of chemicals substances in sex toys. Survey of Chemical Substances in Consumer Products. 2006;77.

[12] Council NR (2009). Phthalates and cumulative risk assessment: the tasks ahead.

[13] Dziobak MK. Characterizing Phthalate Exposure in Common Bottlenose Dolphins (Tursiops truncatus) from Sarasota Bay, Florida 2010–2019. College of Charleston. 2021. 10.3390/ani12070824 PMC8996861 35405813

[14] Peters RJ (2003). Hazardous chemicals in consumer products. TNO report.

[15] SmittenKitten. What’s in your sex toy? Badvibes.org. https://badvibes.org/whats-in-your-sex-toy/. Published 2006. Accessed 8 Feb 2023.

[16] Jiang W, Kim B, Rutka JT, Chan WC (2008). Nanoparticle-mediated cellular response is size-dependent. Nat Nanotechnol.

[17] Gigault J, El Hadri H, Nguyen B, Grassl B, Rowenczyk L, Tufenkji N, Feng S, Wiesner M (2021). Nanoplastics are neither microplastics nor engineered nanoparticles. Nat Nanotechnol.

[18] Tønning K, Hansen PL, Pommer K, Malmgren-Hansen, B. Survey and health assesment of chemicals substances in pleasure gel. https://www2.mst.dk/udgiv/publications/2006/87-7052-220-0/pdf/87-7052-221-9.pdf. Survey of chemical substances in consumer products, Danish EPA 2006, 76.

[19] Sipe JM, Bossa N, Berger W, von Windheim N, Gall K, Wiesner MR. From bottle to microplastics: Can we estimate how our plastic products are breaking down? Science of The Total Environment. 2022;814:152460. 10.1016/j.scitotenv.2021.152460. 10.1016/j.scitotenv.2021.152460 34973311

[20] Cowger W, Steinmetz Z, Gray A, Munno K, Lynch J, Hapich H, Primpke S, De Frond H, Rochman C, Herodotou O (2021). Microplastic spectral classification needs an open source community: open specy to the rescue!. Anal Chem.

[21] Krongauz VV, Ling MT, O’Connell J. Revisiting analysis of phthalate plasticizers concentration in poly (vinyl chloride). J Vinyl Add Tech. 2015;21(3):197–204.

[22] Bossa N, Sipe JM, Berger W, Scott K, Kennedy A, Thomas T, Hendren CO, Wiesner MR (2021). Quantifying Mechanical Abrasion of MWCNT Nanocomposites Used in 3D Printing: Influence of CNT Content on Abrasion Products and Rate of Microplastic Production. Environ Sci Technol.

[23] ECHA(European Chemicals Agency). Restrictions on the manufacture, placing on the market and use of certain dangerous substance, mixtures and articles. https://echa.europa.eu/documents/10162/aaa92146-a005-1dc2-debe-93c80b57c5ee. ANNEX XVII TO REACH – Conditions of restriction.

[24] Thomas T, Review of exposure data and assessments for select dialkyl ortho/phthalates. Rockville : CPSC (Consumer Product Safety Commission) 2010.

[25] Sathyanarayana S, Karr CJ, Lozano P, Brown E, Calafat AM, Liu F, Swan SH (2008). Baby care products: possible sources of infant phthalate exposure. Pediatrics.

[26] Calafat AM. Phthalate Metabolites in Urine. Prevention. https://www.cdc.gov/nchs/data/nhanes/nhanes_09_10/phthte_f_met.pdf. U. C. f. D. C. a., Ed. 2012; Vol. 6306.03.

[27] National Health and Nutrition Examination Survey. Phthalates and Plasticizers Metabolites - Urine (PHTHTE_J). https://wwwn.cdc.gov/Nchs/Nhanes/2017-2018/PHTHTE_J.htm. US Centers for Disease Control and Prevention: August 2021.

[28] Maines R (1989). Socially camouflaged technologies: The case of the electromechanical vibrator. IEEE Technol Soc Mag.

[29] King H (2011). Galen and the widow: towards a history of therapeutic masturbation in ancient gynaecology. EuGeStA: J Gender Stud Antiquity.

[30] Stephanie Osfield W. Your vibrator could be poisoning you. https://nypost.com/2017/04/04/your-vibrator-could-be-poisoning-you/ (Accessed 8 Feb 2023).

[31] Critic, D. L. S. T. Toxic Toys – The Definite Guide to Toxic Sex Toy Awareness. http://dangerouslilly.com/toxictoys/ (Accessed 8 Feb 2023).

[32] Aaronson DS, Shindel AW (2010). Advocating for safer use of sexual enhancement products. J Sex Med.

[33] Dahlberg M, Nordberg M, Pieniowski E, Boström L, Sandblom G, Hallqvist-Everhov Å (2019). Retained sex toys: an increasing and possibly preventable medical condition. Int J Colorectal Dis.

[34] Biesanz Z (2007). Dildos, artificial vaginas, and phthalates: how toxic sex toys illustrate a broader problem for consumer protection. Law & Ineq.

[35] Slovic P, Finucane ML, Peters E, MacGregor DG (2013). Risk as analysis and risk as feelings: Some thoughts about affect, reason, risk and rationality.

[36] Douglas PM, Douglas M. Purity and Danger: An Analysis of Concepts of Pollution and Taboo (1st ed.). Routledge; 1996. 10.4324/9781315015811.

[37] Galaitsi SE, Hendren CO, Trump B, Linkov I (2019). Sex robots—a harbinger for emerging AI risk. Frontiers in Artificial Intelligence.

[38] Shepert, E. 'Bella Dolls' sex doll brothel has opened in Vancouver. https://www.vancouverisawesome.com/courier-archive/news/bella-dolls-sex-doll-brothel-has-opened-in-vancouver-3088481 (Accessed 8 Feb 2023).

