# Supplementary material for: Bringing sex toys out of the dark: exploring unmitigated risks
Source: Microplast nanoplast. 2023 Mar 23;3(1):6. doi: 10.1186/s43591-023-00054-6 (PMC10034881; doi:10.1186/s43591-023-00054-6)
Supplement: Supplementary file 1 — Additional file 1. [file 43591_2023_54_MOESM1_ESM.docx]

Supplementary Materials for

Bringing sex toys out of the dark: exploring unmitigated risks

Joana Marie Sipe, Jaleesia D. Amos, Robert F. Swarthout, Amalia Turner, Mark R. Wiesner, Christine Ogilvie Hendren

Correspondence to: hendrenco@appstate.edu

**This file includes:**

Figs. S1 to S4

Data Figs. S5 to S8

Supplementary Figures

Below in Figures S1 to S3 are Openspecy FTIR results. There is no spectrum for the anal toy because it completely solubilized after extraction.

Figs. S1 to S3 are Openspecy FTIR Results

**S1: Beads OpenSpecy FTIR**


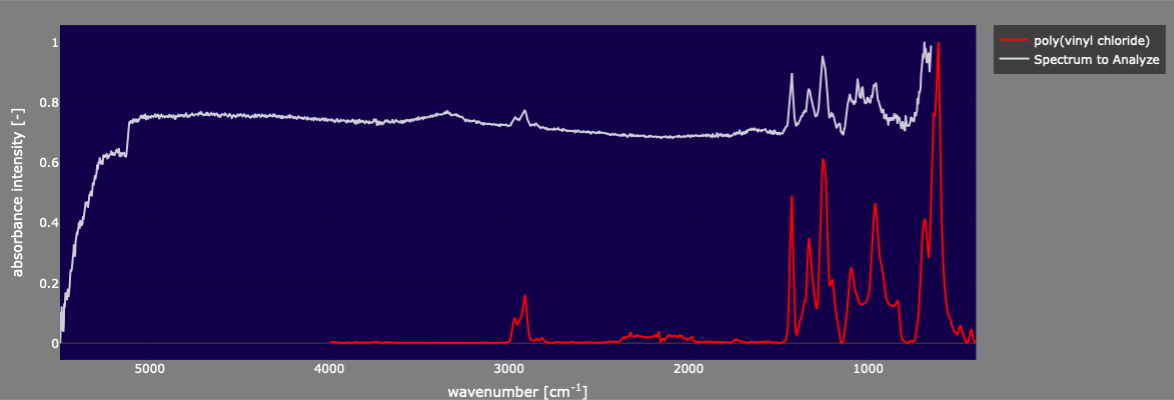


**S2: External Vibrator OpenSpecy FTIR**

**
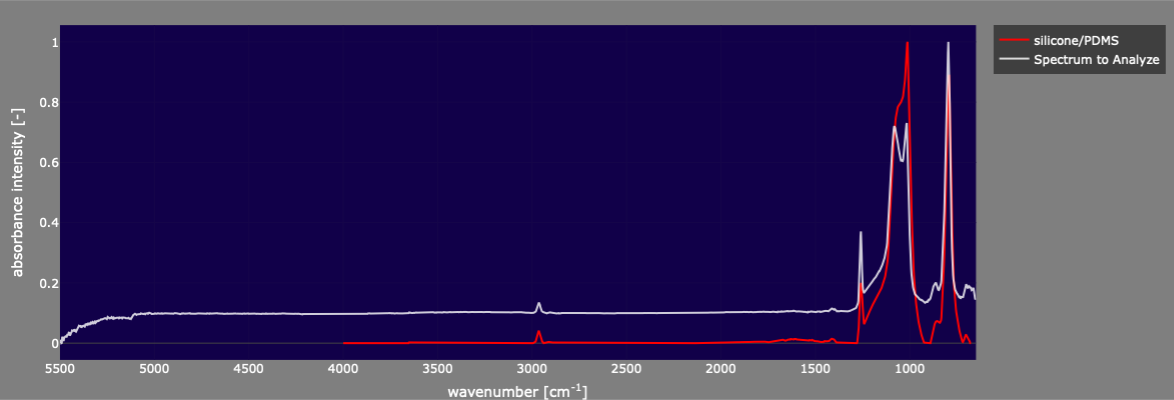
**

**S3: Dual Vibrator OpenSpecy FTIR**

**
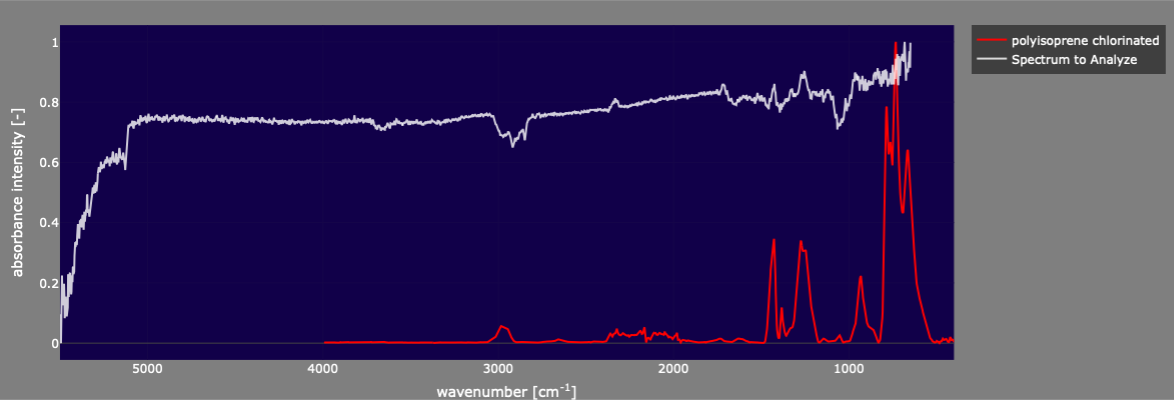
**

**S4: Anal Toy OpenSpecy FTIR**

**
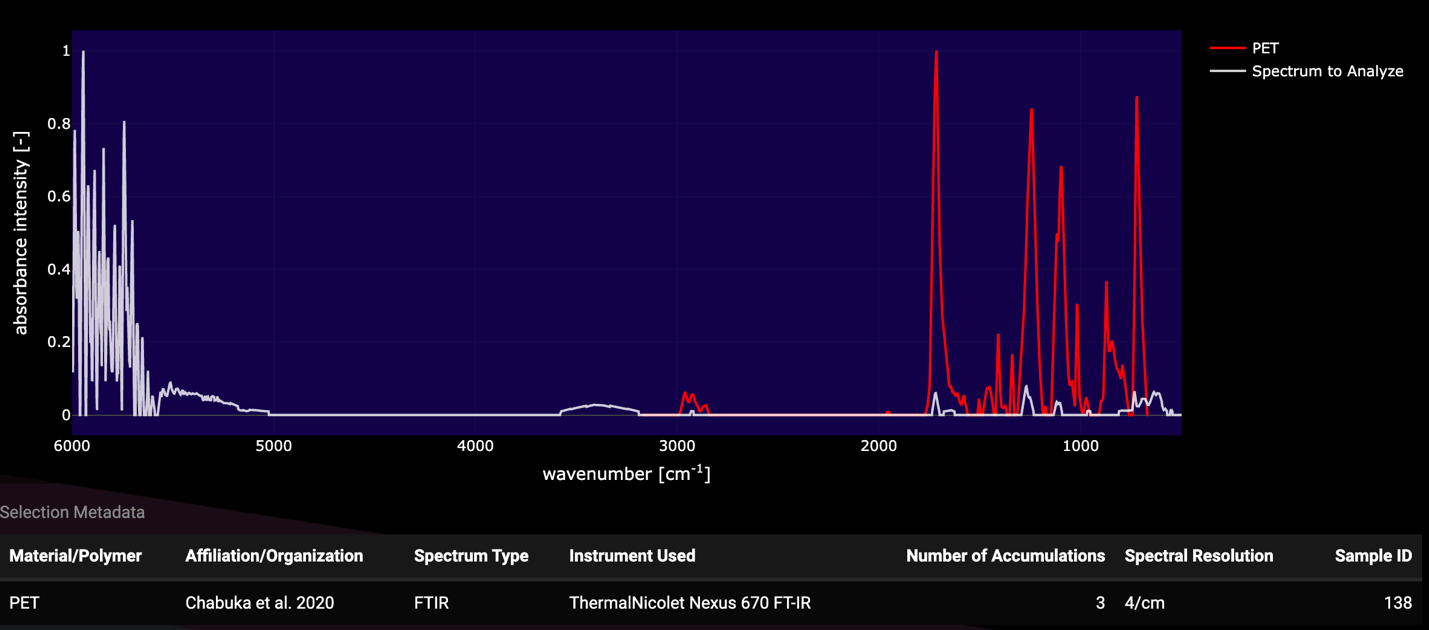
**

Supplementary Data

Below in Figures S5 to S8 are raw files of data used for abrasion quantification.

**S5: Beads Data and Stats File**


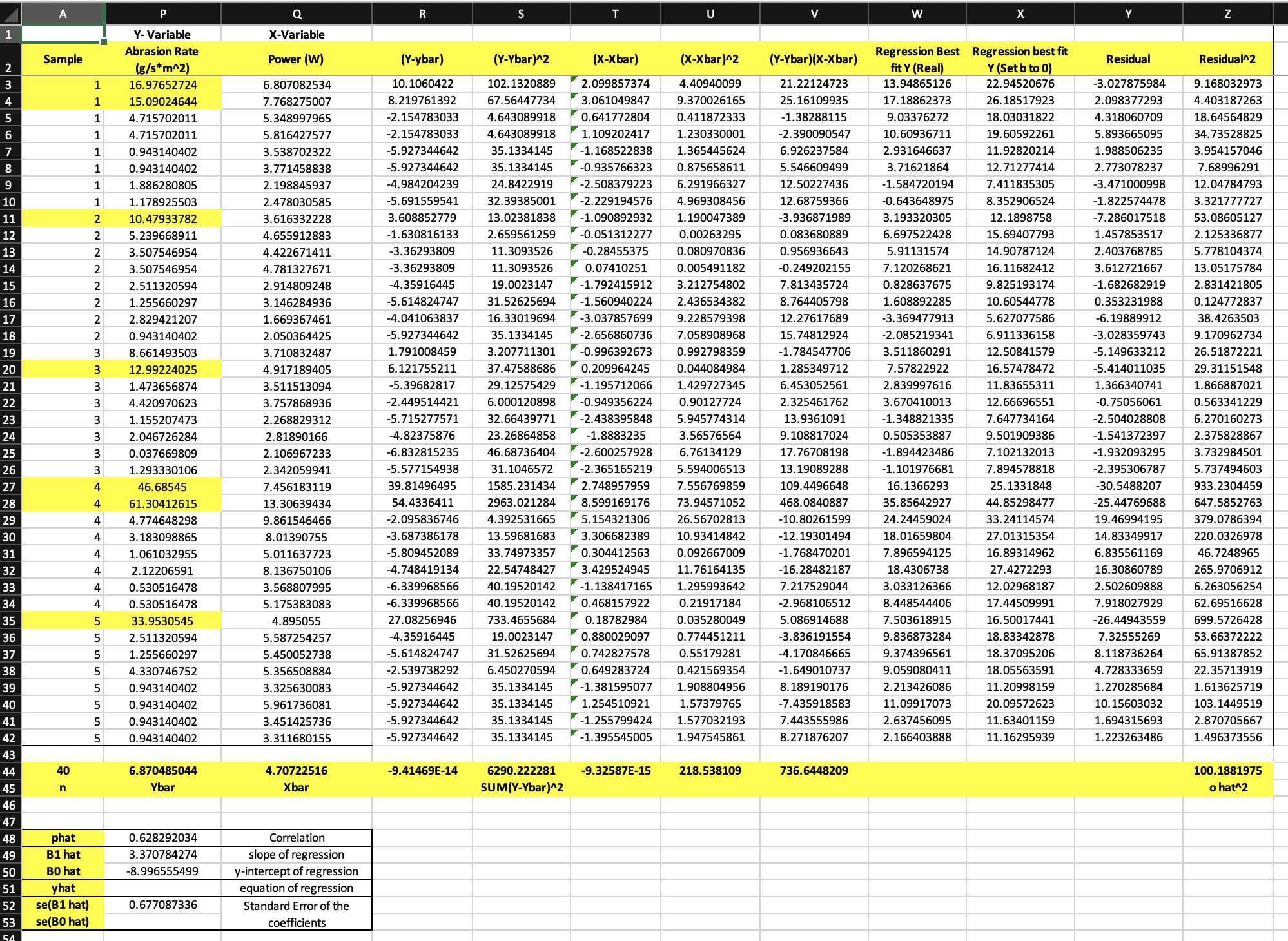


**S6: External Vibrator Data and Stats File**

**
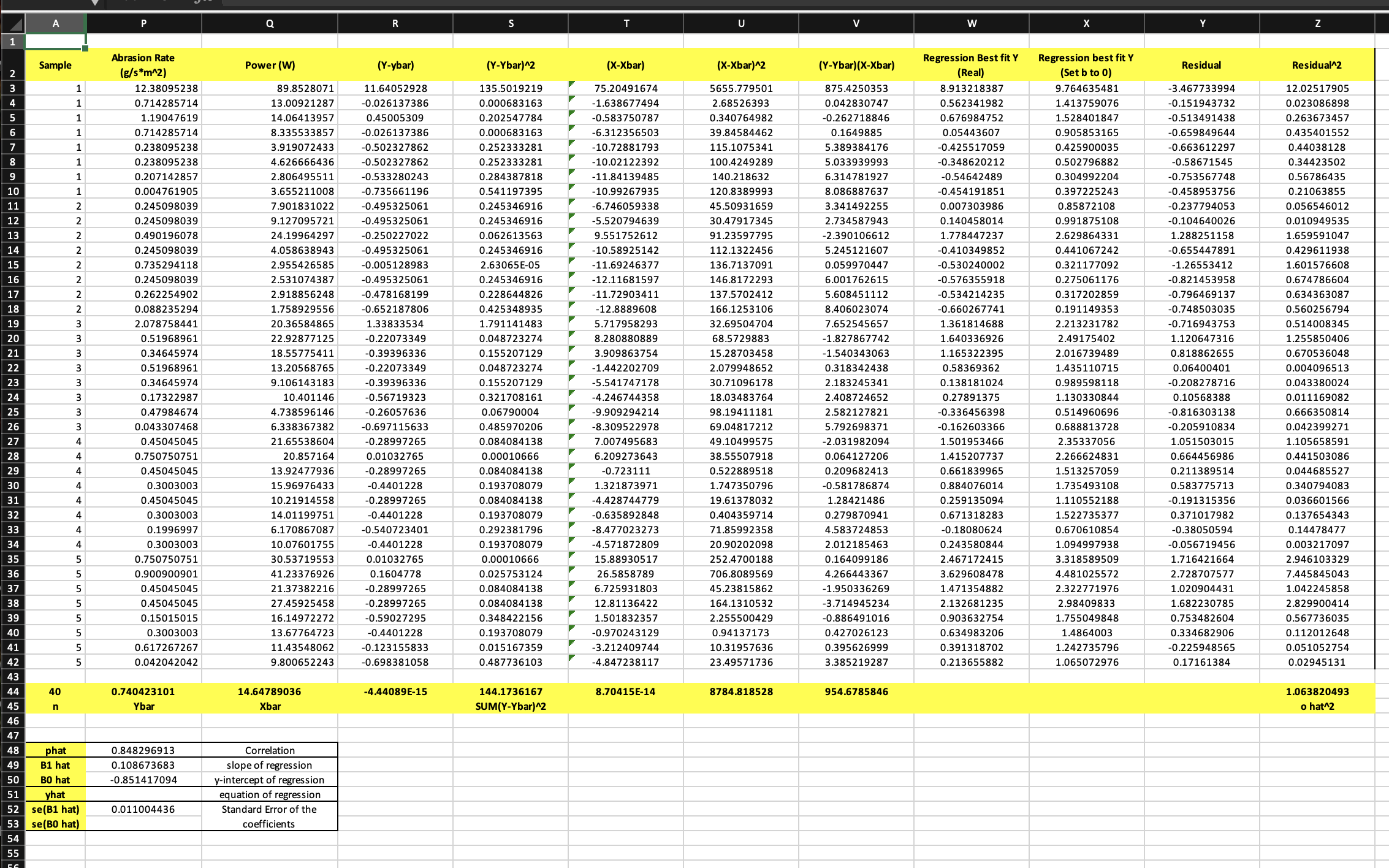
**

**S7: Dual Vibrator Data and Stats File**

**
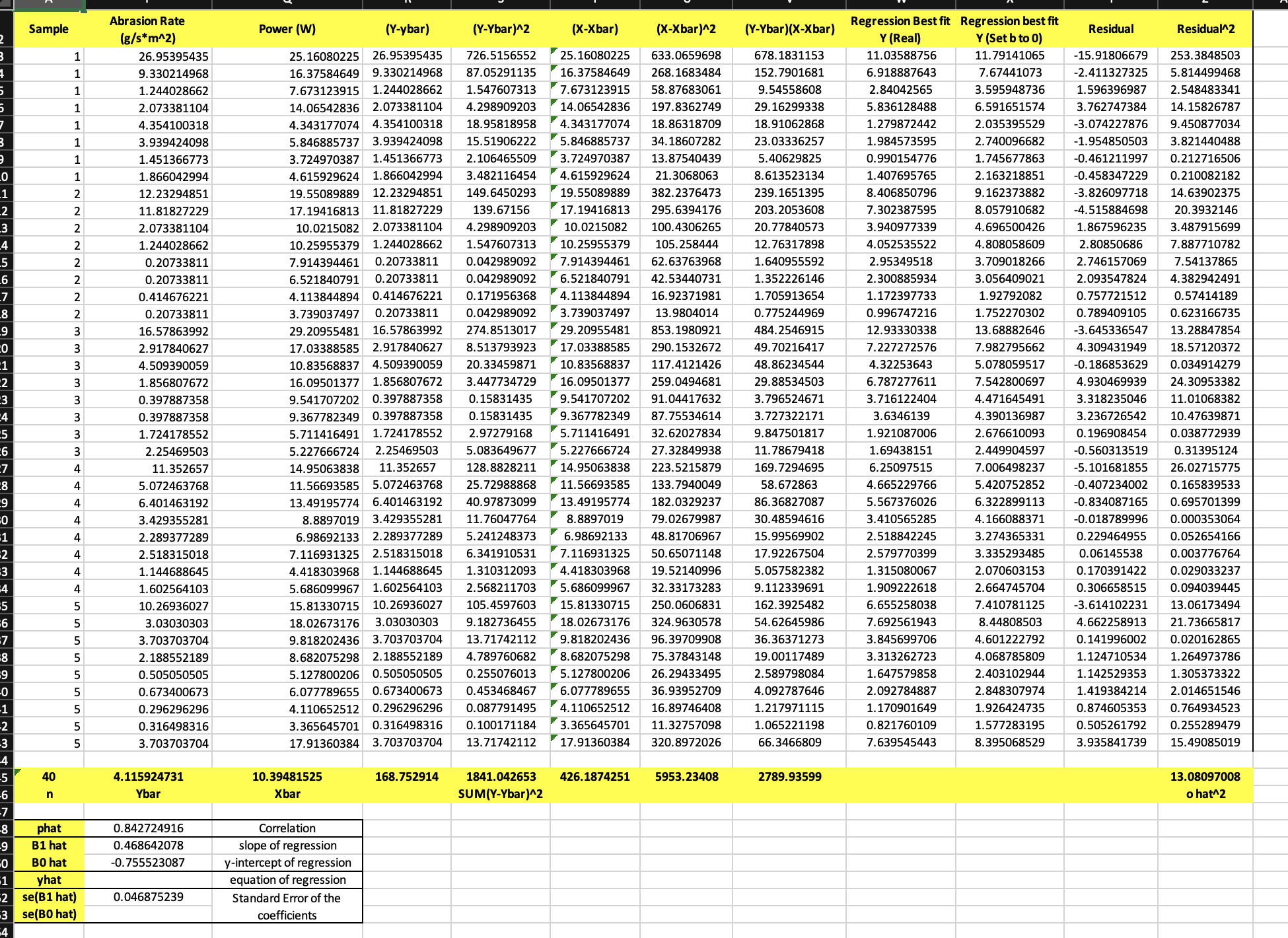
**

**S8: Anal Toy Data and Stats File**

**
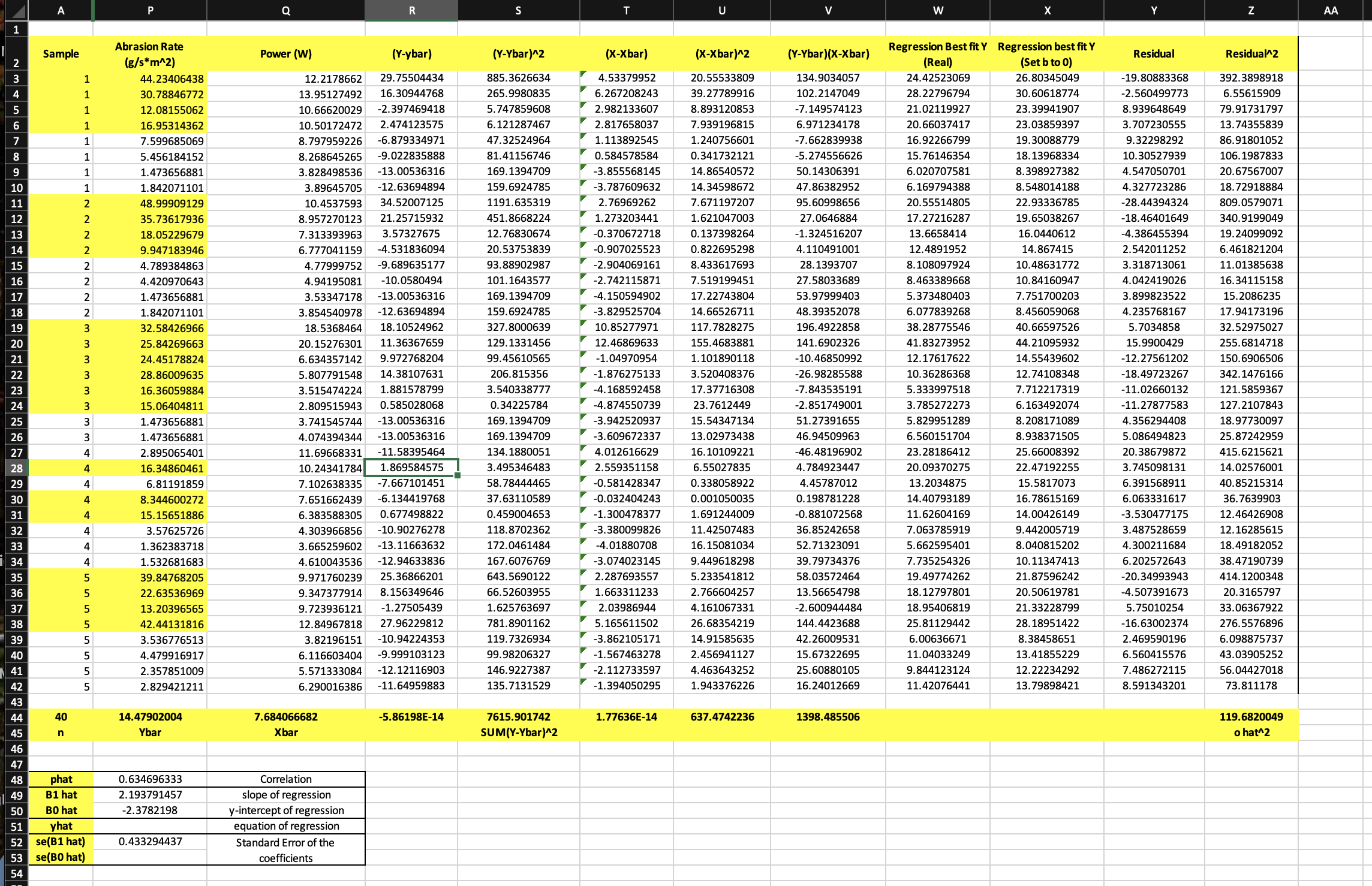
**
